# *Saccharomyces cerevisiae* Eukaryotic Elongation Factor 1A (eEF1A) Is Methylated at Lys-390 by a METTL21-Like Methyltransferase

**DOI:** 10.1371/journal.pone.0131426

**Published:** 2015-06-26

**Authors:** Magnus E. Jakobsson, Erna Davydova, Jędrzej Małecki, Anders Moen, Pål Ø. Falnes

**Affiliations:** Department of Biosciences, Faculty of Mathematics and Natural Sciences, University of Oslo, Oslo, 0316, Norway; Universität Stuttgart, GERMANY

## Abstract

The human methyltransferases (MTases) METTL21A and VCP-KMT (METTL21D) were recently shown to methylate single lysine residues in Hsp70 proteins and in VCP, respectively. The yet uncharacterized MTase encoded by the *YNL024C* gene in *Saccharomyces cerevisiae* shows high sequence similarity to METTL21A and VCP-KMT, as well as to their uncharacterized paralogues METTL21B and METTL21C. Despite being most similar to METTL21A, the Ynl024c protein does not methylate yeast Hsp70 proteins, which were found to be unmethylated on the relevant lysine residue. Eukaryotic translation elongation factor eEF1A in yeast has been reported to contain four methylated lysine residues (Lys30, Lys79, Lys318 and Lys390), and we here show that the *YNL024C* gene is required for methylation of eEF1A at Lys390, the only of these methylations for which the responsible MTase has not yet been identified. Lys390 was found in a partially monomethylated state in wild-type yeast cells but was exclusively unmethylated in a *ynl024cΔ* strain, and over-expression of Ynl024c caused a dramatic increase in Lys390 methylation, with trimethylation becoming the predominant state. Our results demonstrate that Ynl024c is the enzyme responsible for methylation of eEF1A at Lys390, and in accordance with prior naming of similar enzymes, we suggest that Ynl024c is renamed to Efm6 (Elongation factor MTase 6).

## Introduction

Protein methylation is an abundant posttranslational modification, mediated by specific methyltransferases (MTases)^2^ that catalyse the transfer of a methyl group from the methyl donor *S*-adenosylmethionine (AdoMet) to a protein substrate [1,2]. Lysine and arginine residues are the primary targets of methylation, but also other residues such as glutamine and histidine can be methylated [2]. Lysine residues can accept up to three methyl groups, yielding three possible methylation states; mono-, di-, or trimethyllysine. Lysine residues in the N-terminal tails of histone proteins in chromatin are subject to extensive methylation, and a wealth of studies have established that such histone methylations regulate transcriptional activity and chromatin packing, and that the individual modifications exhibit distinct distribution patterns along the genome [1,3]. Besides histones, many other proteins also contain functionally important methylations on lysine residues [3,4].

Based on sequence and structural features, MTases have been categorised in five classes [5]. The largest group of MTases are the so-called seven-beta-strand (7BS) MTases, which contain a characteristic twisted beta sheet consisting of seven strands with a specific topology [6,7]. These enzymes catalyse the methylation of a wide range of substrates, including nucleic acids, lipids, proteins and small metabolites. The second largest MTase class share a defining SET domain (named after the founding members Suv(var)3-9, Enhancer of Zeste [e(z)] and Trithorax) [6,7]. These SET domain enzymes are lysine (K) specific MTases (KMTs) acting on histone tails, and also on various non-histone substrates [8,9]. However, recent studies have revealed that also the 7BS MTases encompass several KMTs, the majority of which target non-histone substrates.

The *S*. *cerevisiae* enzyme Dot1 was the first eukaryotic 7BS KMT to be discovered. Dot1 and its mammalian orthologue DOT1L both catalyse the methylation of Lys79 in the globular domain of histone H3 [10,11]. *S*. *cerevisiae* See1, which later has been renamed Efm4 (Elongation factor MTase 4), and its human orthologue METTL10, both catalyse the dimethylation of eukaryotic translation elongation factor 1A (eEF1A) on Lys318 [12–14]. Very recently, the 7BS MTase encoded by the *YGR001C* gene, which based on bioinformatics had been inferred as an RNA MTase, was somewhat unexpectedly shown to be responsible for trimethylation of Lys79 in eEF1A, and this enzyme was denoted Efm5 [15]. In addition to the 7BS MTases described above, several mammalian and yeast members of the so-called MTase Family 16 (MTF16), a subfamily of the 7BS MTases, have recently been established as KMTs. The *S*. *cerevisiae* MTF16 MTase encoded by the YLR137W gene was shown to monomethylate a Lys residue in the ribosomal protein Rpl1ab [16]. This enzyme, which is only found in fungi, was therefore denoted Rkm5 (Ribosomal protein lysine MTase 5). The *S*. *cerevisiae* protein encoded by the *YBR271W* gene represents another fungi-specific MTF16 enzyme, which was shown to methylate eukaryotic translation elongation factor 2 (eEF2) on Lys613, and, consequently denoted Efm2 [12,17,18]. Furthermore, eEF2 is modified by another MTF16 KMT, which appears to be ubiquitous in the eukaryotic kingdom; the *S*. *cerevisiae* protein encoded by *YJR129C*, which was recently redubbed Efm3, catalyses trimethylation of Lys509 in eEF2, while its human orthologue FAM86A (eEF2-KMT) methylates the corresponding residue, Lys525, in human eEF2 [17–19]. The notion that MTF16 mainly encompasses enzymes with KMT activity has been further strengthened by recent studies on several of the human MTF16 members. It has been demonstrated that these enzymes are highly specific, typically methylating one or two Lys residues in a single substrate (or a group of closely related substrates), and collectively target a broad range of substrates, *i*.*e*. eEF2 (mentioned above), the ATP-dependent chaperone VCP/p97, the DNA repair protein KIN17, calmodulin, the β-subunit of electron transfer flavoprotein and several Hsp70 (HSPA) proteins [19–25]. Somewhat in contrast to these findings, the *S*. *cerevisiae* MTF16 enzyme encoded by the *YIL110W* gene was shown to be required for histidine methylation of the ribosomal protein Rpl3, and this enzyme was named Hmt1p (Histidine specific MTase 1), indicating that the MTF16 enzymes also encompass protein MTases targeting residues other than lysine.

The components of the protein synthesis and folding machinery, such as translation factors, ribosomal proteins and chaperones, are frequently methylated [26]. In several cases, the responsible MTase has been identified; some examples of these were mentioned above. However, for many reported methylations, the identity of the MTase remains elusive. Clearly, methylations on the protein synthesis machinery are functionally important, and ablating the responsible MTases has been shown to cause translation-associated phenotypes, such as decreased accuracy of protein synthesis and increased sensitivity to drugs that inhibit protein synthesis [18,19]. Moreover, it has been demonstrated that the level of some of these methylations is subject to variation between different tissues in mammals or are altered in response to various stimuli, suggesting that they may exert a regulatory function [19,27]. eEF1A, which is a homolog of bacterial EF-Tu, both in terms of sequence and function, is responsible for delivering the aminoacylated tRNA to the ribosome during protein translation, and *S*. *cerevisiae* eEF1A has been shown to contain four methylated lysine residues, Lys30, Lys79, Lys316 and Lys390. As indicated above, two 7BS MTases, Efm4 and Efm5, are responsible for methylation at Lys316 and Lys79, respectively. In addition, Yhl039w, which is a SET enzyme, was shown to mediate methylation at Lys30, and was denoted Efm1 [12,13].

In the present work we have investigated the function of the previously uncharacterized *S*. *cerevisiae* MTF16 member Ynl024c (encoded by gene *YNL024C*), which is the closest yeast homolog of the mammalian Hsp70 (HSPA) specific MTase METTL21A (HSPA-KMT). Somewhat surprisingly, we found that Ynl024c does not target Hsp70 proteins, but rather catalyses the methylation of the eukaryotic elongation factor eEF1A at Lys390, and we suggest that this enzyme is redubbed Efm6 (Elongation factor MTase 6).

## Materials and Methods

### Bioinformatics

NCBI’s BLAST algorithm was used to identify and assess homology between proteins [28]. Alignment of protein sequences was performed with the MUSCLE algorithm embedded in the alignment viewer Jalview [29,30] and prediction of protein secondary structure was performed with Jpred 3 [31]. A structural model of the Ynl024c protein was generated by one-to-one threading using the Phyre server [32]. The PyMOL Molecular Graphics System (Schrodinger) was used for visualization of protein structures.

### Cloning and Mutagenesis

All plasmids used in this study are listed and described in S1 Table. For cloning, open reading frames were amplified with Phusion DNA polymerase (Thermo Scientific) using primers harbouring relevant restriction sites. Amplicons were thereafter cloned into either the bacterial expression vectors pET28a (Novagen) and pGEX-6p (GE Healthcare) or the yeast expression vector pYES260 (Euroscarf) using indicated restriction enzymes (NEB) and T4 DNA ligase (NEB). Site directed mutagenesis was performed using the QuickChange method (Stratagene). All constructs were verified by DNA sequencing.

### Purification of recombinant proteins

Protein purification was essentially performed as previously described [21]. Briefly, bacterial expression plasmids for expression of relevant hexa-histidine (6xHis) or glutathione S-transferase (GST) tagged proteins were transfected into the *Escherichia coli* BL21(DE3)-RIPL (Stratagene) expression strain. Cells were cultured in terrific broth medium at 37°C and 250 rpm. At mid-log phase the temperature was reduced to 16°C and protein expression was induced with isopropyl β-D-1-thiogalactopyranoside (500 μM) and allowed to proceed for 16 h. Cells were harvested by centrifugation and pellets were resuspended in a lysis buffer consisting of 50 mM Tris (pH 8.0), 500 mM NaCl, 10% (w/v) glycerol, 0.5% (w/v) Nonidet P-40, 30 mM imidazole, 3 mM 2-mercaptoethanol, 0.5 mg/ml Lysozyme (Sigma-Aldrich), 25 units/ml Benzonase (Sigma-Aldrich) and cOmplete protease inhibitor tablets (Roche). For GST fusion proteins imidazole was omitted. Lysates were cleared by ultracentrifugation and 6xHis and GST tagged proteins were thereafter purified using Ni-NTA agarose (Qiagen) and Glutathione Sepharose 4B (GE Healthcare), respectively, according to the supplier’s instructions.

### Yeast strains

Yeast BY4742 wild-type (*MAT*α; *his3*Δ*1*; *leu2*Δ*0*; *lys2*Δ*0*; *ura3*Δ*0*) and *ynl024c*Δ (*MAT*α; *his3*Δ*1*; *leu2*Δ*0*; *lys2*Δ*0*; *ura3*Δ*0; ynl024c*::*kanMX4*) strains were acquired from Euroscarf (European Saccharomyces Cerevisiae Archive for Functional Analysis). Ablation of the *YNL024C* gene was confirmed by PCR. Strains for overexpression of 6xHis-tagged Ynl024c were generated by transforming cells with pYES260-*YNL024C* plasmid using the LiAc/SS carrier method [33]. Expression of recombinant protein was induced by shifting the growth medium from YPD (1% Bacto yeast extract, 2% peptone, 2% glucose) to YPG (1% Bacto yeast extract, 2% peptone, 2% galactose). Protein extracts were prepared according to previously established methods [34].

### *In vitro* MTase assays

MTase reactions with purified recombinant proteins were performed at 37°C for 1 h in 50 μl reactions containing assay buffer [50 mM Tris (pH 7.8 at 25°C), 50 mM KCl, 5mM MgCl_2_, 1mM ATP, 13 μM [^3^H]-AdoMet (Perkin Elmer)], 4 μM substrate and 2 μM MTase unless noted otherwise. Reactions were terminated by adding 900 μl ice cold TCA and precipitated material was isolated on a glass fibre filter (Whatman GF/C) using vacuum filtration. Filters were then washed with TCA and absolute ethanol, and incorporated methyl groups were quantified by scintillation counting. For MS analysis radiolabeled AdoMet was replaced by 1.2 mM unlabeled AdoMet (NEB) and MTase reactions were terminated by boiling in NuPAGE buffer (Life Technologies).

MTase reactions with protein extracts as source of enzymatic activity and substrate were performed in the same assay buffer as described above but were instead incubated at 30°C for 1 h. As source of enzymatic activity, 50 μg of a protein extract from *E*. *coli* overexpressing 6xHis-Ynl024c was used, and 50 μg of protein extract from the *ynl024cΔ* yeast strain was used as a source of substrate(s). Proteins were then separated by SDS-PAGE and transferred to a PVDF membrane. The membrane was stained with Ponceau S, treated with the scintillation enhancer EN^3^HANCE and exposed to Carestream Kodak BioMax MS film (Sigma-Aldrich) for 8 weeks at -80°C.

### Mass spectrometric analysis

Liquid chromatography (LC) coupled to tandem mass spectrometry (MS/MS) analysis was performed as described previously [21]. In brief, protein samples were separated by SDS-PAGE, whereafter in-gel proteolysis of relevant gel regions was performed using either trypsin (Sigma-Aldrich), Asp-N (Roche) or Arg-C (Roche). After chromatographic separation, peptides were analysed with a LTQ-Orbitrap XL mass spectrometer (Thermo Scientific) using collision induced dissociation. MS data was analysed with SEQUEST, using in-house maintained databases of the *S*. *cerevisiae* proteome or eEF1A sequences from various organisms. The following modifications were selected for analysis: methionine oxidation as well as mono-, di- and tri-methylation of lysine.

Chromatograms for qualitative comparative analysis of lysine methylation states were generated by gating for m/z ratios of the various methylated forms of relevant peptides. The y-axis for the discrete methylated forms was then normalized with respect to signal intensity.

The fractional occupancy of individual lysine methylation states for peptides was determined as the area under the chromatogram corresponding to a unique modification state divided by the sum of corresponding areas for all modification states. Areas under chromatograms were determined by integration using Qual Browser (v2.0.7).

## Results

### Bioinformatics and structural analysis of Ynl024c

We and others have previously unravelled the function of the human KMTs METTL21A and METTL21D, showing that they target Hsp70 (HSPA) proteins and VCP, and we therefore redubbed them HSPA-KMT and VCP-KMT, respectively (however, as METTL21A still represents the official gene name, it will be used here) [20–22]. These enzymes comprise, together with METTL21B and METTL21C, a small group of four closely related human MTases, representing a subset of the ten human MTF16 enzymes [22]. In *S*. *cerevisiae*, the uncharacterized putative MTase Ynl024c represents the closest sequence homolog of the METTL21 proteins, and we therefore set out to characterize this enzyme. BLAST searches, using Ynl024c as query, revealed that, among human proteins, METTL21A is the closest sequence homolog of Ynl024c (Table 1). Ynl024c also shows sequence similarity to *S*. *cerevisiae* Efm2, but it is more similar to the human METTL21 enzymes.

**Table 1:** Closest homologs of Ynl024c in *S*. *cerevisiae* and *H*. *sapiens*.

Organism	Protein*	Expect value	Accession #
*S*. *cerevisiae*	Efm2	7e-7	NP_009830.3
*H*. *sapiens*	METTL21A	**2e-17**	NP_660323.3
	METTL21D	3e-14	NP_078834.2
	METTL21B	8e-11	NP_056248.2
	METTL21C	3e-9	NP_001010977.1

*Identified by BLAST search versus non-redundant proteins sequences from all organisms using Ynl024c (NP_014374.1) as query.

Hits with e-value < 1e-5 were considered to be homologs.

Conversely, BLAST searches using the human METTL21 proteins as queries, all yielded Ynl024c as the primary *S*. *cerevisiae* hit, but also identified Efm2 and the uncharacterized protein Ylr285w as homologs (Table 2) (Ylr285w is also annotated as a nicotinamide N-MTase 1, Nnt1, but this is likely a misnomer, given its similarity to MTF16 proteins).

**Table 2:** Closest homologs of human METTL21 proteins in *S*. *cerevisiae*. *

Query		Identified homolog (e-value)**		
Protein	Accession #	Ynl024c (NP_014374.1)	Efm2 (NP_009830.3)	Ylr285w (NP_013387.1)
METTL21A	NP_660323.3	**2e-17**		5e-6
METTL21D	NP_078834.2	**3e-14**	1e-7	
METTL21B	NP_056248.2	**8e-11**	7e-7	8e-6
METTL21C	NP_001010977.1	**3e-9**		5e-6

*Identified by BLAST versus non-redundant sequences from all organisms whereafter hits were filtered for *S*. *cerevisiae* S288c (taxid: 559292).

**Hits with e-value < 1e-5 considered to be homologs. Best hit is indicated in bold.

**Fig 1:**
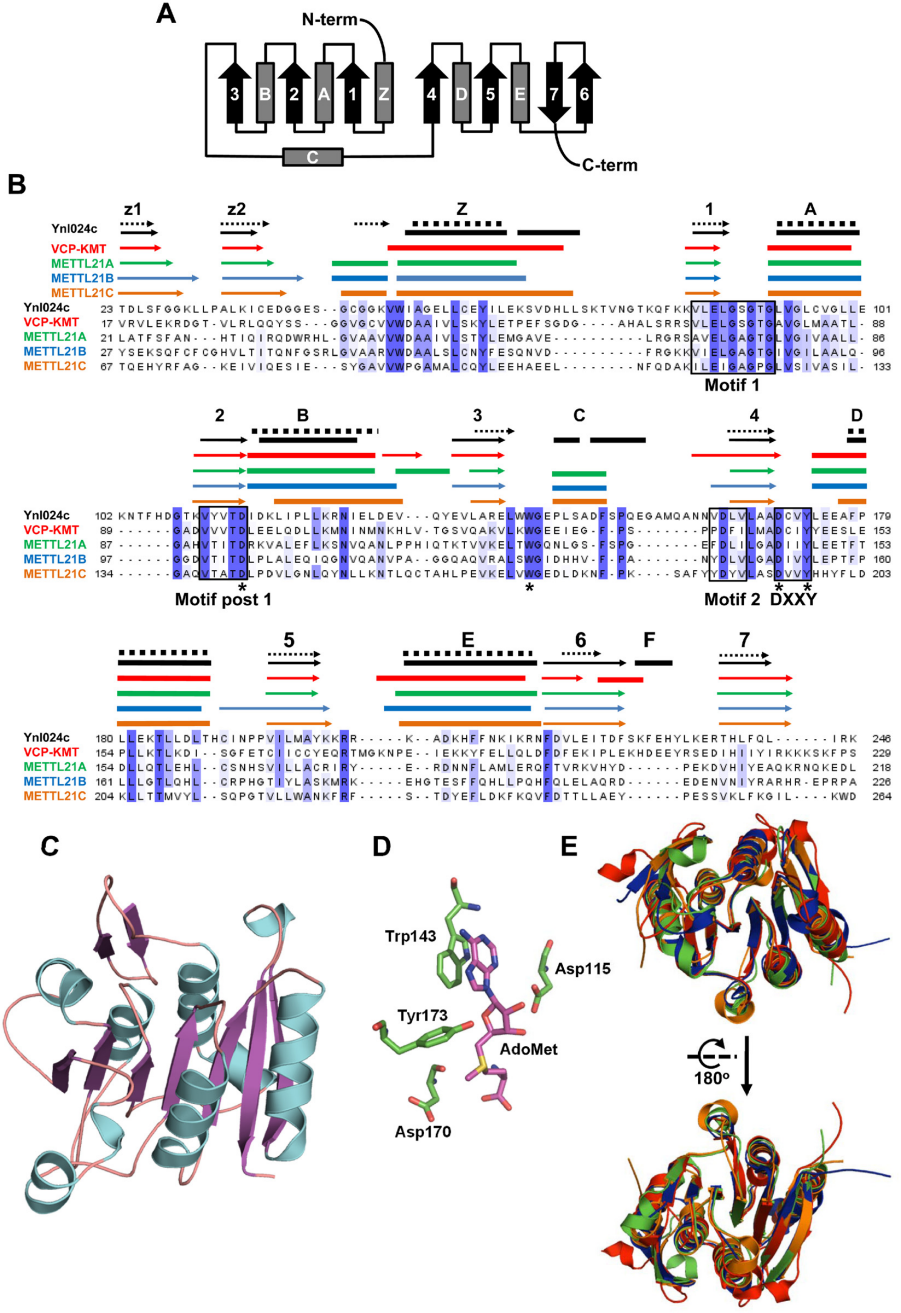
Sequence analysis and structural modelling of Ynl024c. (A) Topology diagram of the canonical 7BS MTase fold. Arrows and rectangles indicate β-strands and α-helices, respectively. The seven β-strands are designated “1–7”, the α-helices connecting them “A-F”, and the secondary structure elements preceding the 7BS-fold denoted “Z”. B, Protein sequence alignment of Ynl024c and human METTL21 proteins. Motifs “I”, “Post I” and “II”, which are shared by all 7BS MTases, as well as the DXXY motif, a hallmark of MTF16, are indicated by boxes. Above the alignment are indicated the secondary structure elements from the solved crystal structures of VCP-KMT (red; pdb 4LG1), METTL21A (green; pdb 4LEC), METTL21B (blue; pdb 4QPN) and METTL21C (orange; pdb 4MTL), a predicted Ynl024c structure (black; see also (C)), as well as a secondary structure prediction for Ynl024c, performed with Jpred 3 (dashed, black). β-strands and α-helices are indicated by arrows and thick lines, respectively, and the numbering/lettering of these are as outlined in A). Asterisks indicate conserved active site residues represented in (D). (C) Predicted structural model of Ynl024c. The model was generated by one-to-one threading with Phyre2, using METTL21A as a template. (D) localization of putatively important catalytic residues (green) in the active site of the Ynl024c structural model. AdoMet is shown in purple. The shown residues are indicated by asterisk in the sequence alignment in (B). (E) Structural alignment of human VCP-KMT, METTL21A, METTL21B and METTL21C (Color code as in (B)).

The 7BS MTases show an archetypical topology (Fig 1A) and several characteristic sequence motifs. An alignment of Ynl024c with the human METTL21 proteins clearly demonstrates that Ynl024c harbours hallmark conserved motifs found in 7BS MTases, *i*. *e*. Motif I, Motif post I and Motif II (Fig 1B). Similar to other MTF16 members, Ynl024c possesses the characteristic DXXY [(D/E)-X-X-(Y/F)] motif located downstream of Motif II (Fig 1B).

Recently, the structures of all four human METTL21 proteins were released in the Protein Data Bank, and we have used this information to infer structural characteristics of the Ynl024c protein. We made a structural model of Ynl024c using METTL21A as a template, performed by one-to-one threading with Phyre2 (Fig 1C and 1D) [32]. We next mapped all the secondary structure elements from this model, as well as from solved structures of the four METTL21 proteins, onto the sequence alignment, and this clearly demonstrated an almost identical organisation of secondary structure elements (Fig 1B). We also made a secondary structure prediction for Ynl024c using Jpred 3 [31], and this prediction agreed well with the predicted structural model. The Ynl024c structural model shows, similar to the METTL21 structures, a canonical 7BS topology, as depicted in Fig 1A, preceded by two additional β-strands, z1 and z2 (Fig 1B and 1C). In this model, the conserved residues Asp170 and Tyr173 of the DXXY motif are positioned in the vicinity of the methyl group donated by AdoMet in the methylation reaction, suggesting that they are catalytically important (Fig 1D). Trp143, which is conserved in the MTF16 enzymes, appears to stack with the adenine base of AdoMet (Fig 1D). Asp115 corresponds to a conserved (as Glu or Asp), catalytically important residue found in Motif Post 1 of all 7BS enzymes, shown to hydrogen bond with the-OH groups on the ribose moiety in AdoMet, and the position of this residue in the Ynl024c structural model is clearly compatible with such a role. Importantly, a structural alignment of the four human METTL21 proteins shows a high level of similarity (Fig 1E), and the sequence similarity between some of the METTL21 enzymes is lower than that between METTL21A and Ynl024c, suggesting that the modelled structure of Ynl024c likely represents a close approximation of the “true” structure (Fig 1E). In conclusion, this initial bioinformatics analysis firmly establishes Ynl024c as a MTase showing a high degree of structural and sequence similarity to the human METTL21 enzymes, in particular to METTL21A, whilst showing less similarity to other yeast MTases.

### Investigating Hsp70 proteins as potential Ynl024c substrates

**Fig 2:**
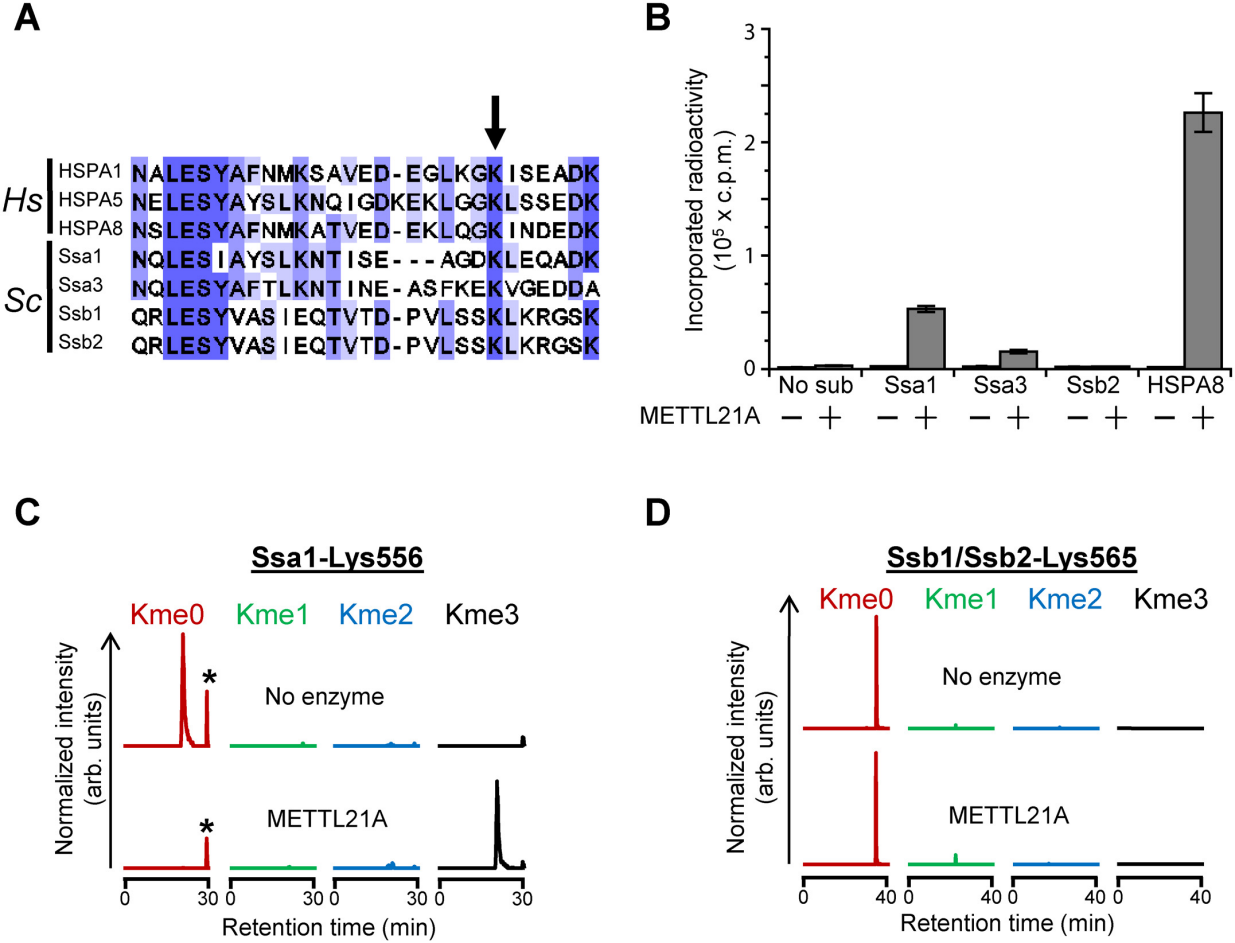
Evaluating Ynl024c as a functional homolog of the Hsp70-specific METTL21A. (A) Protein sequence alignment of various Hsp70 proteins from *H*. *sapiens* (*Hs*) and *S*. *cerevisiae* (*Sc*) showing the region surrounding the lysine targeted by METTL21A (arrow). Shown proteins are: HSPA1(P08107), HSPA5(P11021), HSPA8(P11021), Ssa1(P10591), Ssa3(P09435), Ssb1(P11484) and Ssb2(P40150). (B) Activity of METTL21A on recombinant yeast Hsp70 proteins. Recombinant METTL21A was incubated with the indicated recombinant *E*. *coli*-expressed yeast Hsp70 proteins, as well as with human HSPA8, in the presence of [^3^H]AdoMet, and the amount of TCA-insoluble radioactivity measured by scintillation counting. Error bars indicate standard deviation (n = 3). (C) Lys556 in Ssa1 is not methylated *in vivo*. Yeast whole cell extract (WCE) (20 μg) was incubated in the absence (upper) or presence (lower) of METTL21A (100 pmol). MS chromatograms of Asp-N generated peptides encompassing residues D555-A560 of Ssa1 (as shown in (E) and (F)), gated for m/z corresponding to peptides with un-, mono-, di- and trimethylated Lys556. Peaks corresponding to an unrelated peptide with a m/z-ratio matching the unmethylated species of the peptide are indicated by asterisks. (D) Lys565 in Ssb1/Ssb2 is not methylated *in vivo*. Same as in (C), except that the analysed peptide corresponds to L546-R568 from Ssb1/Ssb2 and was generated by cleavage with Arg-C. Tandem mass spectra of relevant peptides are shown in S1 Fig.

We have previously reported that Ynl024c is not a functional homolog of VCP-KMT/METTL21D, which trimethylates Lys315 in VCP, as the corresponding lysine residue is unmethylated in Cdc48, the yeast orthologue of VCP [22]. However, as Ynl024c is more similar to the Hsp70-specific MTase METTL21A (HSPA-KMT), we set out to investigate whether Ynl024c targets Hsp70 proteins. Human METTL21A has been reported to trimethylate Lys561 in the constitutively expressed Hsp70 protein HSC70/HSPA8 as well as the corresponding residue in several other human Hsp70/HSPA proteins [20,21]. A sequence alignment of various Hsp70 proteins from *H*. *sapiens* and *S*. *cerevisiae* demonstrates that a lysine residue is present at the corresponding position in several of the main Hsp70s in *S*. *cerevisiae*, *e*.*g*. SSA1, SSA3, SSB1, and SSB2 (the latter two are highly similar, showing >99% sequence identity) (Fig 2A). Initially, we therefore set out to test whether recombinant yeast Hsp70 proteins could be methylated by recombinant Ynl024c, but we were unfortunately unable to express and purify soluble, recombinant Ynl024c protein. However, we found that the yeast Hsp70 proteins SSA1 and SSA3, but not SSB2, could be methylated by human METTL21A *in vitro*, thus suggesting that yeast HSP70 proteins have the potential to become methylated by a METTL21A-like enzyme (Fig 2B). To further investigate the possibility that Ynl024c catalyses methylation of Hsp70 proteins, we analysed the methylation state of *S*. *cerevisiae* Hsp70s *in vivo*. To this end, an endoprotease digest of yeast proteins excised from the Hsp70-containing region of a SDS-PAGE gel was analysed by mass spectrometry. In this analysis, peptides covering the relevant Lys residue in SSA1 (Lys556) and SSB1/SSB2 (Lys565) were exclusively detected as unmethylated in wild-type cells (Fig 2C, 2D and S1 Fig). To exclude the possibility that our inability to detect methylated SSA1 was due to technical problems, we also investigated the methylation status of SSA1 in a yeast extract incubated with human METTL21A, and in this case SSA1 was found almost exclusively in the trimethylated state (Fig 2C). These results demonstrate that yeast Hsp70 proteins are unmethylated at the Lys residue corresponding to Lys561 in human HSPA8, and, consequently, that Ynl024c is likely not a functional orthologue of human METTL21A.

### Identification of methylated lysines in S. cerevisiae eEF1A

As Ynl024c did not appear to be involved in Hsp70 methylation, we started searching for other Ynl024c substrates. We have previously identified unique substrates for related enzymes using recombinant MTase either as bait in tandem affinity purification or as a source of enzyme in activity-based global substrate screens [19,21,24]. However, as we were unable to purify recombinant Ynl024c we instead turned to a candidate based approach for substrate identification. For several reasons, we found eEF1A to be an interesting candidate: i) eEF1A has been reported to be methylated on several lysine residues, and, when the present study was initiated, the MTase responsible for several of these methylations was unknown; ii) eEF1A is, similarly to Hsp70s and VCP (the substrates of its closest mammalian homologs, METTL21A and VCP-KMT) a highly abundant, nucleotide-binding protein; iii) the closest yeast homolog of Ynl024c, Efm2, was recently found to methylate Lys residues in another elongation factor, eEF2.

To investigate whether Ynl024c is involved in any of the reported methylations on eEF1A, the lysine methylation status of eEF1A-derived tryptic peptides, either from wild-type yeast or from a *YNL024C* deletion strain (*ynl024cΔ*), was investigated by MS. In this analysis, we qualitatively confirmed all previously reported lysine methylation sites (*i*.*e*. Lys30, Lys79, Lys316 and Lys390) in eEF1A (Table 3). Interestingly, Lys390 was exclusively detected as unmethylated in the *ynl024c* strain whereas peptides covering the same residue were detected as mono- and/or di-methylated in wild-type cells, suggesting that methylation of this residue is dependent on Ynl024c.

**Table 3:** Lysine methylated peptides in eEF1A in wild type and Ynl024c deficient yeast.

Peptide sequence*	Position	Modified residue	Modification
STTTGHLIY**K**CGGIDK	21–36	30	mono
GITIDIALW**K**FETPK	70–84	79	di/tri
NVSV**K**EIR	312–319	316	di/tri
KLEDHP**K**FLK	384–393	390	mono/di**
KLEDHP**K**	384–390	390	mono**

*Methylated residue indicated in bold.

**Exclusively detected in wild type cells.

### Ynl024c mediates methylation of Lys390 in eEF1A *in vivo*

**Fig 3:**
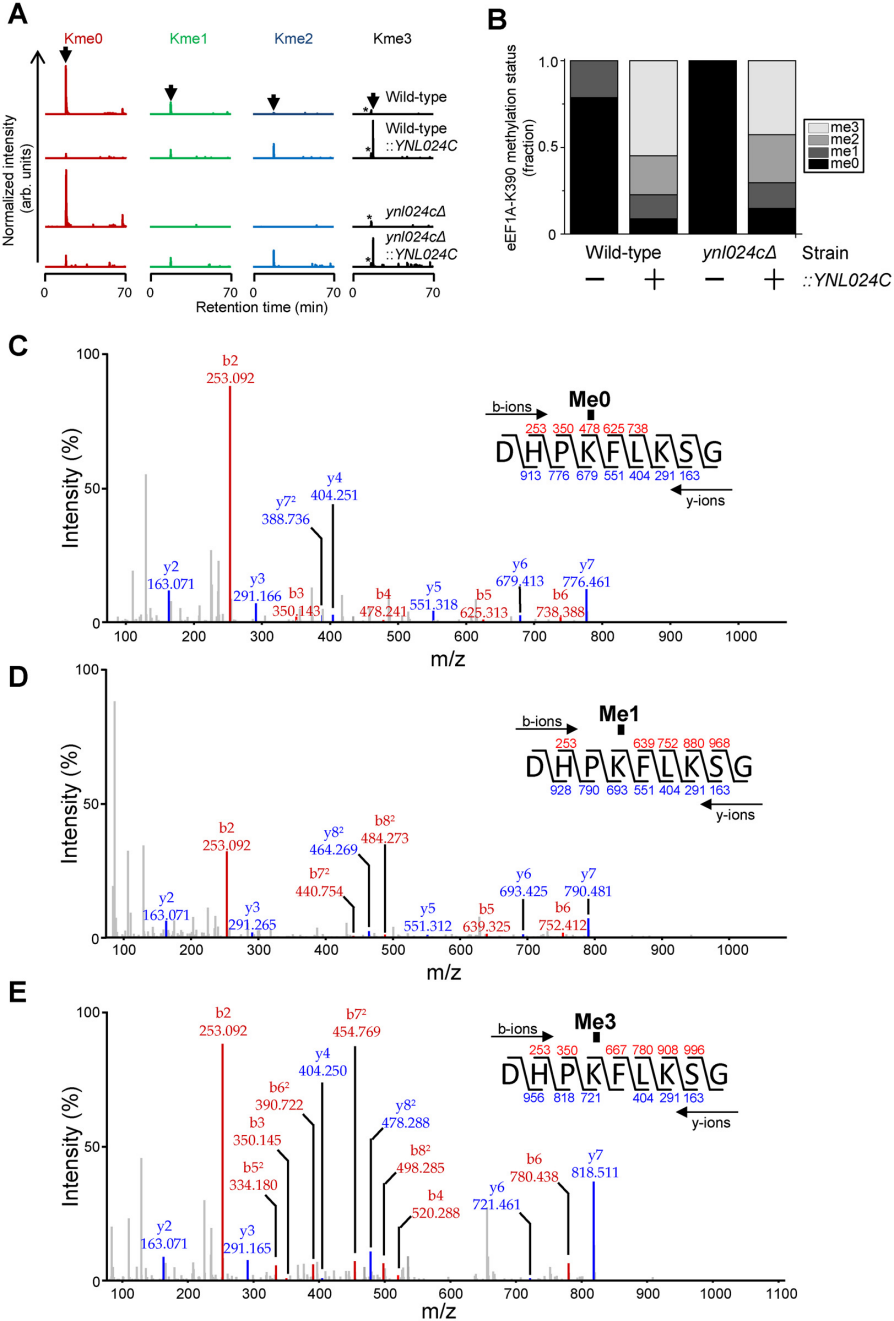
Ynl024c-mediated methylation of eEF1A *in vivo*. (A) Qualitative analysis of Ynl024c-dependent methylation of Lys390 in eEF1A. MS chromatograms gated for the different lysine methylation states of Asp-N generated peptides corresponding to aa 387–395 of *S*. *cerevisiae* eEF1A from wild-type and Ynl024c deficient (*ynl024cΔ*) yeast or these strains with overexpression of Ynl024c, denoted “Wild-type::*YNL024C*”or “*ynl024cΔ*::*YNL024C*“, respectively. The expected elution time for the relevant peptide is indicated by an arrow above the upper trace. The peak corresponding to an unrelated peptide with a m/z-ratio matching the trimethylated peptide species is indicated by an asterisk. (B) Quantitative analysis of Ynl024c-dependent methylation of Lys390 in eEF1A. Quantitative representation of the data from (A). The fractional occupancy of the various lysine methylation states was determined as the relative signal for corresponding peptides in (A), determined by integration. C-E, MS/MS fragmentation pattern supporting the identity of analysed peptides. Representative annotated spectra for un- (C), mono- (D) and trimethylated (E) peptides corresponding to peaks in (A) are shown. Spectra for the dimethylated peptide is shown in S2 Fig.

Our inability to detect of methylation of eEF1A at Lys390 in the *ynl024cΔ* yeast suggested Ynl024c as the MTase responsible for this modification. To further investigate this, we set out to analyse the relative abundance of the various methylated forms of Lys390, and to address the potential effect of Ynl024c over-expression. We used the endoprotease Asp-N to generate a Lys390-encompassing peptide for relative quantification of the different methylated forms by MS, since trypsin-mediated cleavage is inhibited by lysine methylation, and therefore introduces a methylation-dependent bias in peptide generation [35,36]. These experiments revealed that, although Lys390 in eEF1A is primarily found in the unmethylated state in wild-type cells, a considerable portion (~20%) is monomethylated at this residue (Fig 3A–3D). In contrast, Lys390 in eEF1A from *ynl024cΔ* cells was exclusively detected in the unmethylated state (Fig 3A–3C).

Interestingly, when Ynl024c was overexpressed, either in wild-type or *ynl024cΔ* cells, the di- and tri-methylated forms of Lys390 in eEF1A were the dominant species (>70% of total) (Fig 3A, 3B and 3E; S2 Fig). In addition, the lysine in Ssa1/Ssb1/Ssb2 that correspond to the target site of human METTL21A in Hsp70 was exclusively found as unmethylated in cells overexpressing Ynl024c (S3 Fig), demonstrating that the enzyme is highly specific for eEF1A. Furthermore, the degree of methylation at Lys390 observed in the Ynl024c over-expressing strains was slightly higher in the wild-type than in the *ynl024cΔ* strain (~2.2 vs ~2.0 modifications per substrate), likely reflecting an additive contribution both from ectopically expressed and endogenous Ynl024c. Taken together, these data demonstrate that *in vivo* methylation of Lys390 in eEF1A is strictly dependent on Ynl024c and that the degree of methylation can be modulated by altering the level of this MTase.

### Ynl024c catalyses methylation of eEF1A *in vitro*

**Fig 4:**
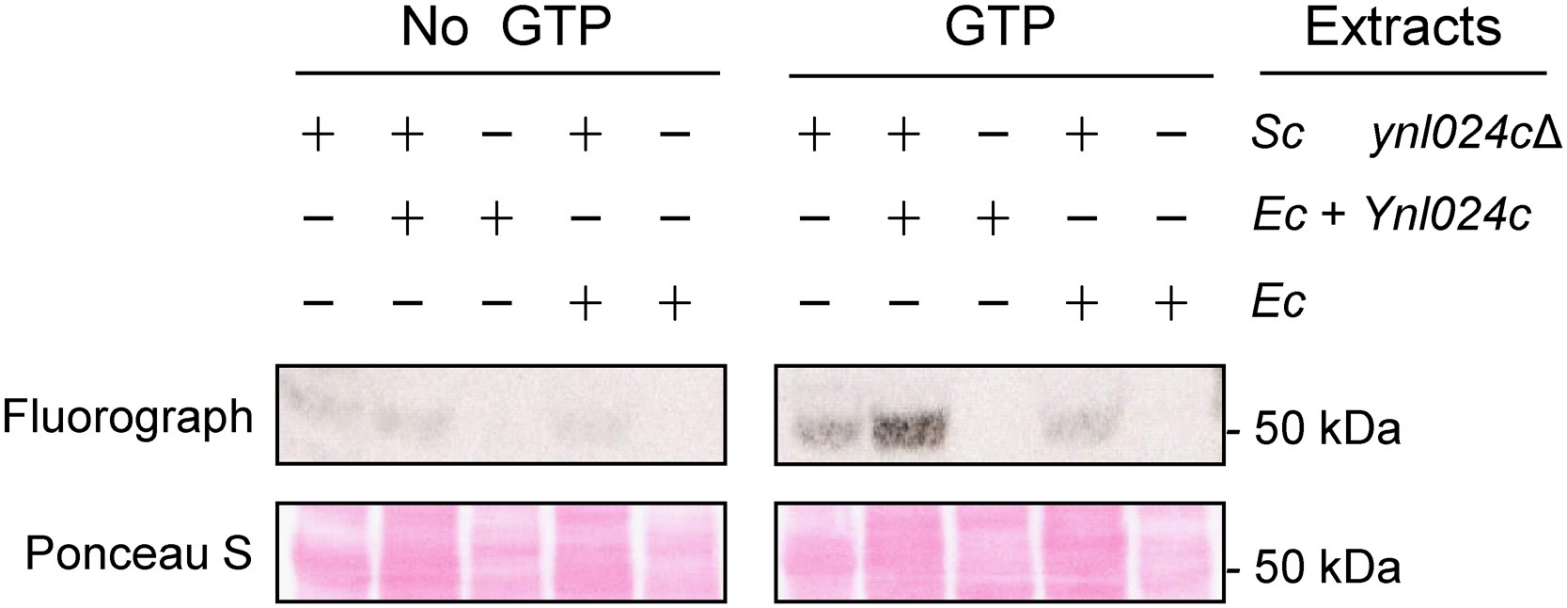
*In vitro* MTase activity of Ynl024c. Protein extract from the *ynl024cΔ* yeast strain (denoted “*Sc ynl024c*Δ”) was treated with a protein extract from *E*. *coli* either expressing 6xHis-tagged Ynl024c from the pET28a-*YNL024C* plasmid (denoted “*Ec*+ Ynl024c”) or devoid of an expression plasmid (denoted “*Ec*”), at 37°C for 60 min in the presence of [^3^H]AdoMet, and 1 mM GTP where indicated. Proteins were separated by SDS-PAGE, transferred to a PVDF membrane and then subjected to Ponceau S staining (bottom) and fluorograpy (top).

As our efforts to purify soluble recombinant Ynl024c from *E*. *coli* had proven unsuccessful, we investigated whether a crude protein extract from an *E*. *coli* strain overexpressing Ynl024c would manifest the anticipated eEF1A-methylating activity *in vitro*. To this end, such an extract was incubated with a *ynl024cΔ* yeast extract in the presence of [^3^H]AdoMet. Proteins were then separated by SDS-PAGE, and incorporation of [^3^H]methyl groups was detected by fluorography. eEF1A is a GTP-binding protein, and since we previously observed that the addition of ATP enhances the methylation of HSPA8 by human METTL21A [21], we also investigated the effect of GTP addition on Ynl024c-mediated methylation of eEF1A *in vitro*. In the absence of GTP, we observed only very weak labelling of the ~50 kDa region corresponding to the molecular weight of eEF1A (Fig 4). This weak activity was not influenced by the presence of the Ynl024c *E*. *coli* extract, suggesting that it may reflect eEF1A methylation by one of the other eEF1A-specific yeast MTases present in the yeast extract. In contrast, when GTP was present, a markedly stronger, Ynl024c-specific labelling was observed in the 50 kDa region of the *ynl024cΔ* yeast extract (Fig 4). Taken together, these results suggest that the Ynl024c protein is capable of directly methylating eEF1A, and that the observed effects of *YNL024C* deletion or over-expression on eEF1A methylation are not indirect, but reflect KMT activity of Ynl024c on eEF1A.

### Lys392 in mammalian eEF1A is not methylated

**Fig 5:**
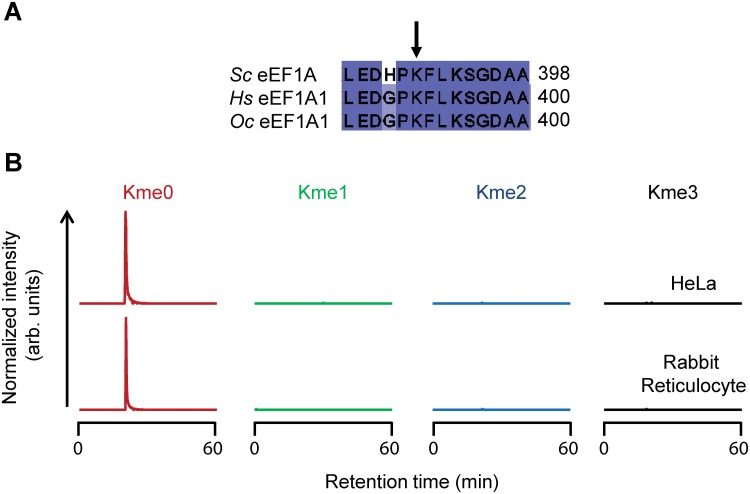
Methylation status of eEF1A in mammalian cells. (A) Alignment of various eEF1A sequences from *Homo sapiens* (*Hs*), *Oryctolagus cuniculus* (*Oc*; rabbit) and *Saccharomyces cerevisiae* (*Sc*), showing the region surrounding the lysine targeted by Ynl024c (arrow). Shown proteins are *Hs* eEF1A1 (NP_001393.1), *Oc* eEF1A1 (NP_001075808.1) and *Sc* eEF1A (NP_009676.1). (B) Methylation status of eEF1A1 in HeLa cells and in rabbit reticulocytes. Chromatograms gated for the different lysine methylation states of Asp-N generated peptides corresponding to aa 389–397 in human and rabbit eEF1A1. Tandem mass spectra supporting the identity of analyzed peptides are shown in S4 Fig.

Having firmly established that Lys390 in eEF1A is methylated by Ynl024c in *S*. *cerevisiae*, we considered the possibility that one of the human METTL21 proteins (or another MTase) could perform the same methylation on mammalian eEF1A. eEF1A is extremely well conserved among eukaryotes, and a sequence alignment shows that the mammalian protein indeed contains a lysine residue at the position corresponding to Lys390 in the yeast sequence (Fig 5A). We analyzed the methylation status of this residue (Lys392) in protein extracts from rabbit (*O*. *cuniculus*) reticulocytes, a commonly used system for *in vitro* translation, as well as in human HeLa cells (Fig 5 and S4 Fig). Lys392 was exclusively detected as unmethylated in these experiments, indicating that enzymatic methylation of this residue is not conserved within eukaryotes, and that mammalian METTL21 enzymes do not target this site.

## Discussion

In the present study, we demonstrate that the previously uncharacterized putative MTase Ynl024c is responsible for one of the four reported lysine methylations on *S*. *cerevisiae* eEF1A, namely of Lys390. With that, all four MTases responsible for these methyl modifications have now been identified, as Efm1, Efm5 and Efm4 were shown to methylate eEF1A on Lys30, Lys79 and Lys316, respectively [12,13,15]. Based on the previous naming of elongation factor-specific MTases in yeast, we suggest that Ynl024c is renamed Efm6.

Compared with other sites targeted by MTF16 enzymes, the level of methylation at Lys390 is very low. The only observed methylation state was monomethylation, found only on ~20% of the eEF1A molecules, and similarly, a recent study reported a fractional occupancy of only ~5% for monomethylation at Lys390 [37]. Beyond translation, eEF1A is involved in several other cellular processes, and, conceivably, distinct eEF1A subpopulations with differing PTM patterns may be specialized towards the various eEF1A functions, and the subpopulation of eEF1A methylated on Lys390 may be involved in one of the non-canonical functions. Another and not mutually exclusive possibility is that eEF1A methylation is subject to active regulation. In response to various stimuli, the cell may alter Lys390 methylation status through regulating the activity or expression of Efm6 and/or lysine-specific demethylases (KDMs); the latter type of enzymes play important roles in regulating the methylation levels of histones. Indeed, we found that increasing the expression of Efm6 dramatically increased eEF1A methylation levels, showing that the entire cellular eEF1A pool is available for methylation, and that the overall Lys390 methylation levels can readily be modulated.

Regulation of protein synthesis through post-translational modification of translation factors is an important part of several adaptive cellular responses; much-studied examples are GCN2-mediated phosphorylation of eukaryotic initiation factor 2 (eIF2) and phosphorylation of eEF2 by eEF2 kinase [38,39]. Less is known about regulation of eEF1A, but it has been shown that eEF1A can be targeted by various kinases, including Raf, which was shown to regulate the half-life of mammalian eEF1A [40]. Regarding methylation of eEF1A, it has been demonstrated that simultaneous mutations of the four lysine methylation sites to arginine does not affect the proliferation rate of the resulting mutant yeast strain nor the enzymatic properties of the eEF1A protein *in vitro* [41]. However, the methylation status of eEF1A is subject to modulation *in vivo*, at least under certain circumstances, as sporulation is accompanied by a dramatic increase in eEF1A methylation in the fungus *Mucor racemosus*, concomitant with an increase in eEF1A activity [42].

We were unable to purify recombinant soluble Efm6 protein showing activity on eEF1A. However, co-incubation of extracts from *E*. *coli* over-expressing recombinant Efm6 with *efm6Δ* yeast in the presence of [^3^H]AdoMet and GTP, led to the labelling of a ~50 kDa protein, indicating that Efm6 is indeed capable of directly methylating eEF1A. It should be noted however, that proteins that are part of a larger complex, such as eEF1A, may be methylated prior to complex assembly, and become inaccessible for methylation once part of the complex. For example, some of the studies revealing MTases responsible for ribosomal protein methylation have not been able to demonstrate a direct activity of the enzyme on the substrate [43,44], and we have previously shown that formation of the VCP homohexamer prevents methylation of VCP by VCP-KMT [22].

**Fig 6:**
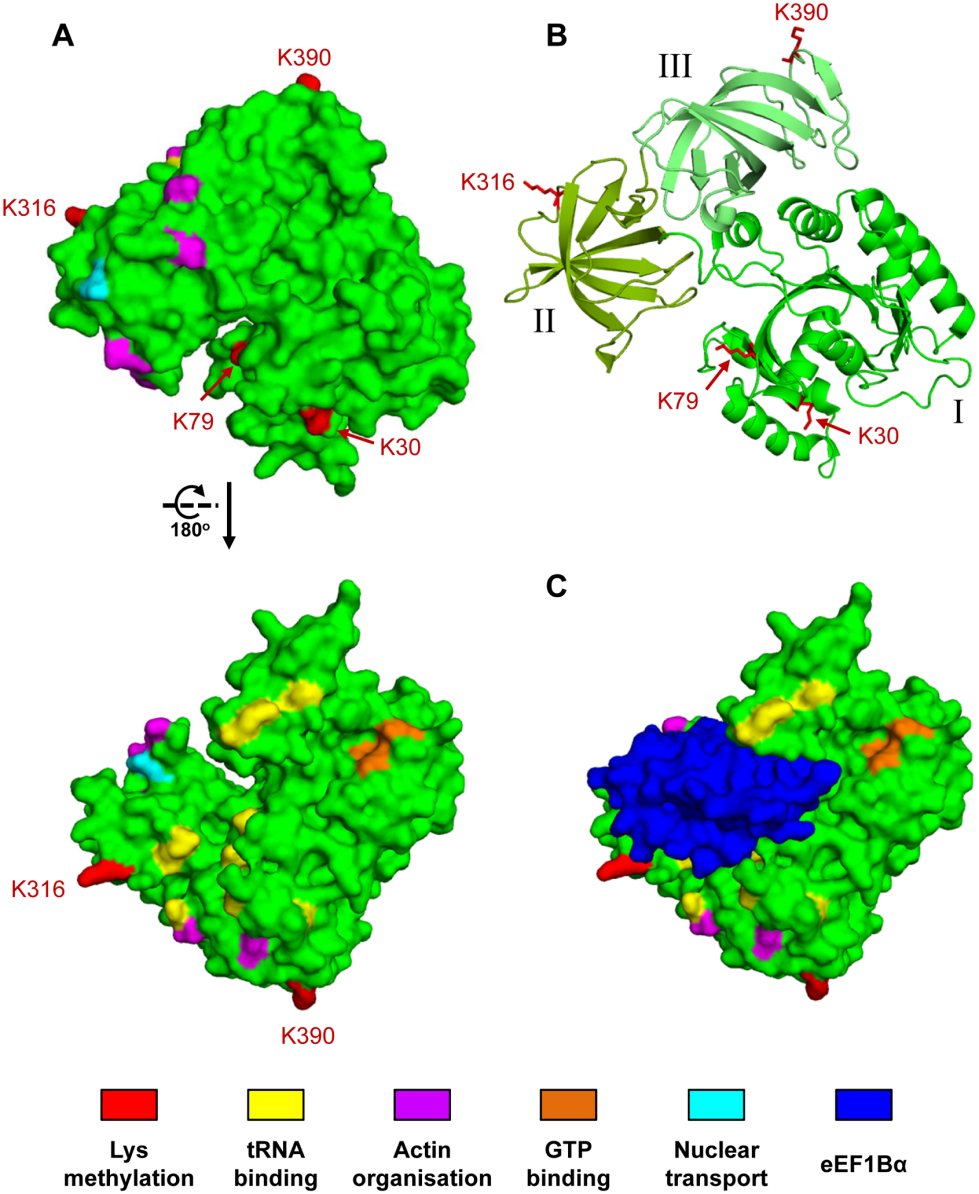
Visualisation of methylation sites and functionally important residues on the *S*. *cerevisiae* eEF1A three-dimensional structure. The structures were generated from PDB 1IJE [47]. (A) Surface representation of eEF1A. Red (and labelled), Lys methylation sites (Lys30, Lys 79, Lys316 and Lys390); yellow, residues that, based on information from bacterial Ef-Tu, are predicted to interact with tRNA (Lys62, Arg69, Lys100, Asn101, Glu291, Arg320, His347, Lys406, Arg425) [45,46]; purple, residues implicated in actin binding and bundling (Asn305, Phe308, Asn309, Ser405) [48–50]; orange, residues involved in GTP binding (Asn153, Lys154, Met155, Asp156) [51]; cyan, residues involved in nuclear transport [50,52]. (B) the structure shown in (A) (upper panel) in ribbon representation. The three domains I, II, and III, that constitute eEF1A are indicated in distinct shades of green. (C) The eEF1A/eEF1B**α** complex. The eEF1B**α** subunit (in blue) is indicated on the structure shown in the upper panel in (A).

The functional significance of eEF1A methylation remains largely elusive. As eEF1A is an extensively studied protein, many of the regions and residues involved in various aspects of eEF1A function have been identified. To obtain an indication of the possible function of Efm6-mediated methylation of eEF1A on Lys390, we have marked all four Lys methylation sites on the eEF1A structure, as well as the residues important for different eEF1A functions, such as actin interaction, nuclear transport, GTP-binding and tRNA binding (Fig 6). As no three-dimensional structure of a complex between tRNA and eEF1A is available, putative tRNA-binding residues were identified from corresponding information on the bacterial counterpart EF-Tu [45,46]. Residues that have been shown to influence the tRNA binding of EF-Tu, and that are conserved between EF-Tu and eEF1A, were characterized as putatively tRNA binding. This analysis did not reveal any striking co-localization of Lys390, nor of any of the other methylated lysine residues (Lys30, Lys79, Lys316) in the vicinity of any of the residues implicated in eEF1A function (Fig 6). Thus, the precise function of Efm6-mediated eEF1A methylation does remain an enigma and further studies are required to reveal its role.

## Supporting Information

S1 Fig MS/MS fragmentation patterns of peptides analyzed in Fig 1. (A-C) Annotated mass spectra with detected b- and y-ions indicated for the unmethylated (A) and trimethylated (B) peptide covering D555-A560 in Ssa1, as well as for the unmethylated peptide encompassing L546-R568 in Ssb1/Ssb2 (C) are shown.
(TIF)

S2 Fig MS/MS fragmentation pattern of eEF1A derived peptide dimethylated at Lys390. Detected b- and y-ions from peptide corresponding to aa 387–395 of eEF1A are indicated in red and blue, respectively.
(TIF)

S3 Fig Ynl024c specifically methylates eEF1A-Lys390 in vivo. (A-B) Yeast HSP70 proteins are not methylated upon over-expression of YNL024C. MS chromatograms gated for the various methylated forms of peptides corresponding to (A) aa D555-A560 in Ssa1 and (B) aa L546-R568 in Ssb1/Ssb2. *C*, Methylation status of eEF1A-Lys390 upon over-expression of YNL024C. MS chromatograms gated for the different methylated forms of peptides corresponding to aa 387–395 in eEF1A1.
(TIF)

S4 Fig MS/MS fragmentation pattern supporting lack of Lys392 methylation in eEF1A1 in mammalian cells. Annotated mass spectra from unmethylated peptide corresponding to aa 389–397 in human (A) and rabbit (B) eEF1A1.
(TIF)

S1 Table Plasmid constructs generated for and used in present study. (DOCX)
