# Supplementary figures and images for: Saccharomyces cerevisiae Eukaryotic Elongation Factor 1A (eEF1A) Is Methylated at Lys-390 by a METTL21-Like Methyltransferase
Source: PLoS One. 2015 Jun 26;10(6):e0131426. doi: 10.1371/journal.pone.0131426 (PMC4482628; doi:10.1371/journal.pone.0131426)

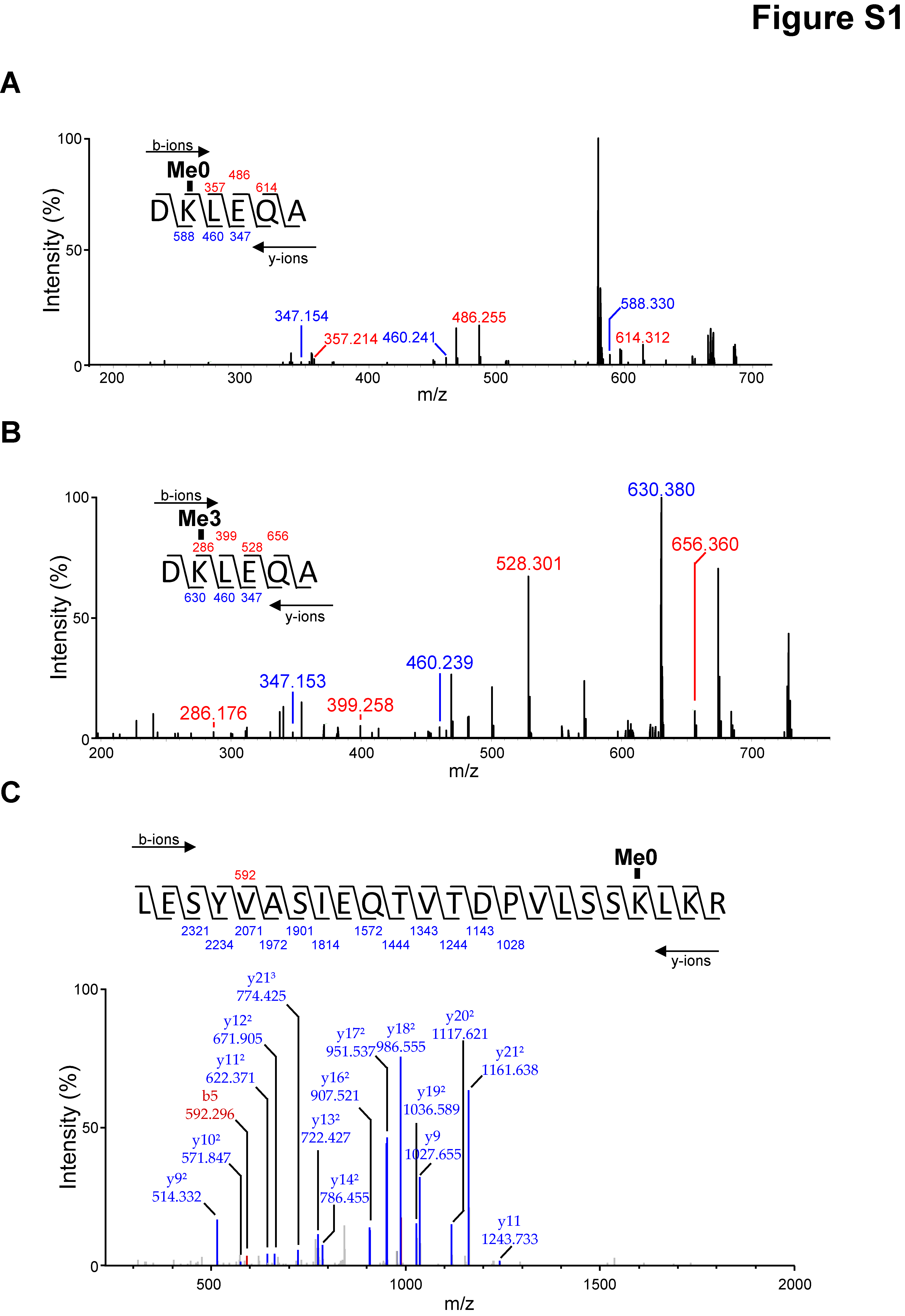

Supplement: S1 Fig — (A-C) Annotated mass spectra with detected b- and y-ions indicated for the unmethylated (A) and trimethylated (B) peptide covering D555-A560 in Ssa1, as well as for the unmethylated peptide encompassing L546-R568 in Ssb1/Ssb2 (C) are shown. (TIF) [file pone.0131426.s001.tif]

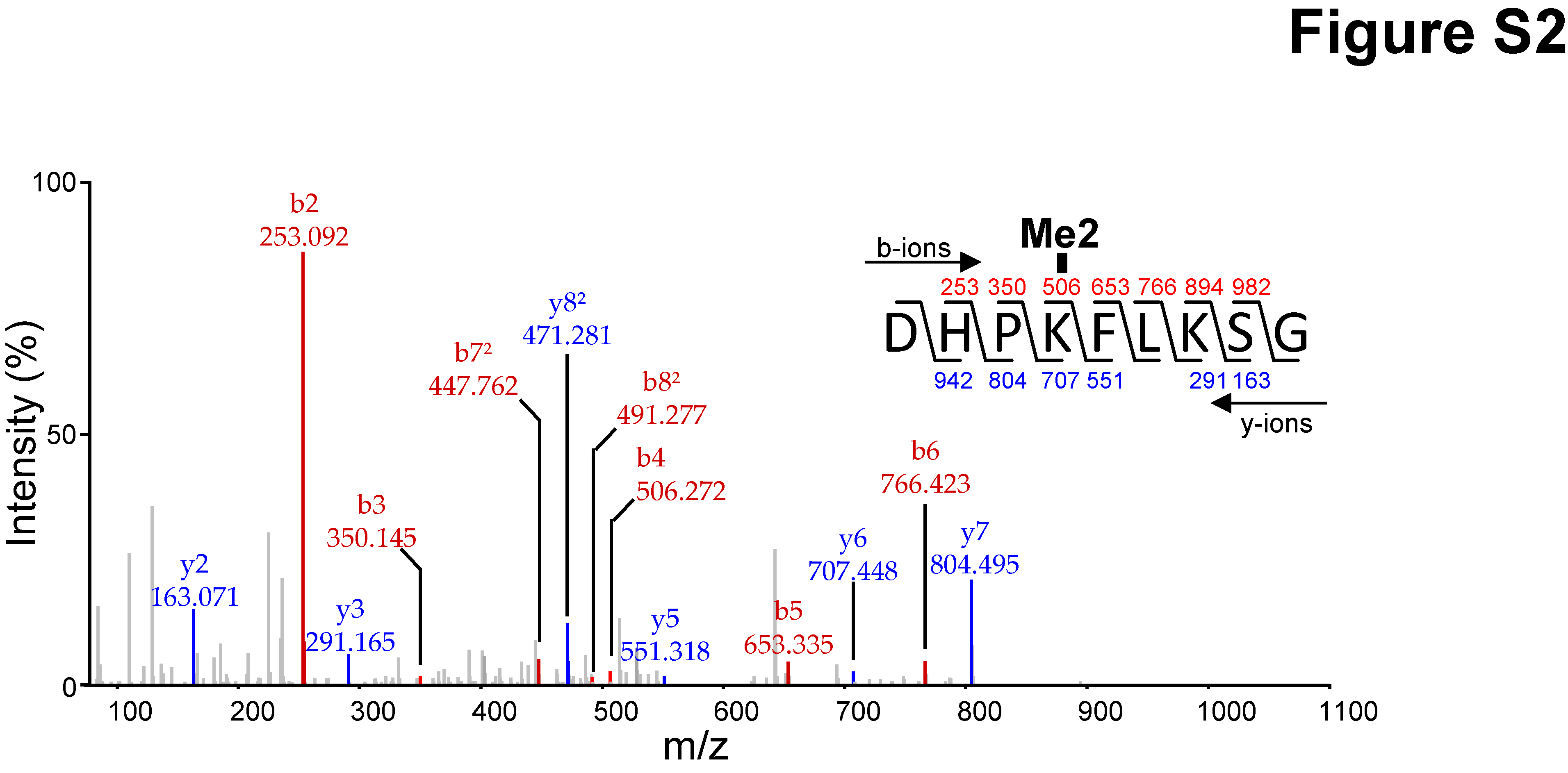

Supplement: S2 Fig — Detected b- and y-ions from peptide corresponding to aa 387–395 of eEF1A are indicated in red and blue, respectively. (TIF) [file pone.0131426.s002.tif]

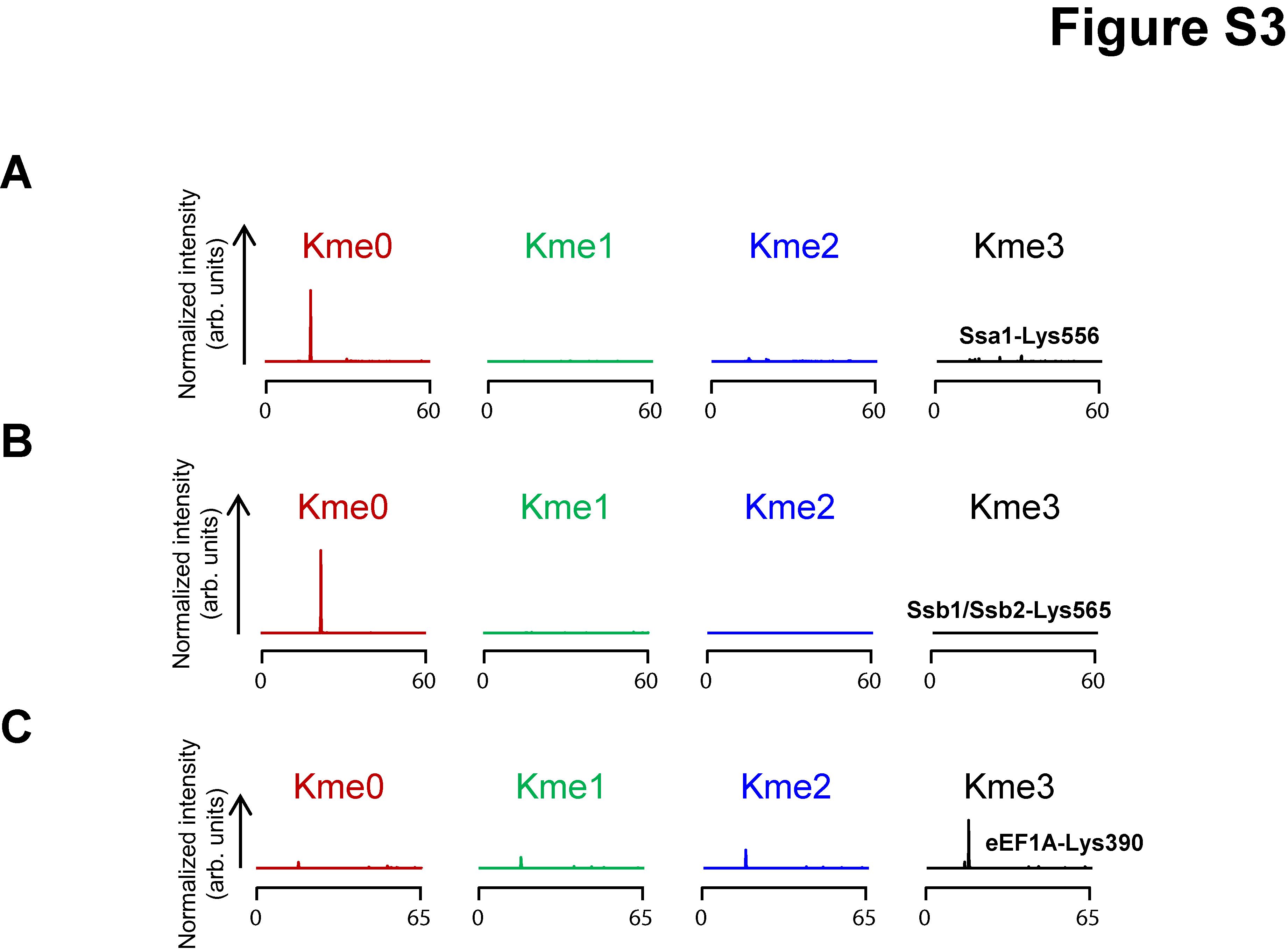

Supplement: S3 Fig — (A-B) Yeast HSP70 proteins are not methylated upon over-expression of YNL024C. MS chromatograms gated for the various methylated forms of peptides corresponding to (A) aa D555-A560 in Ssa1 and (B) aa L546-R568 in Ssb1/Ssb2. C, Methylation status of eEF1A-Lys390 upon over-expression of YNL024C. MS chromatograms gated for the different methylated forms of peptides corresponding to aa 387–395 in eEF1A1. (TIF) [file pone.0131426.s003.tif]

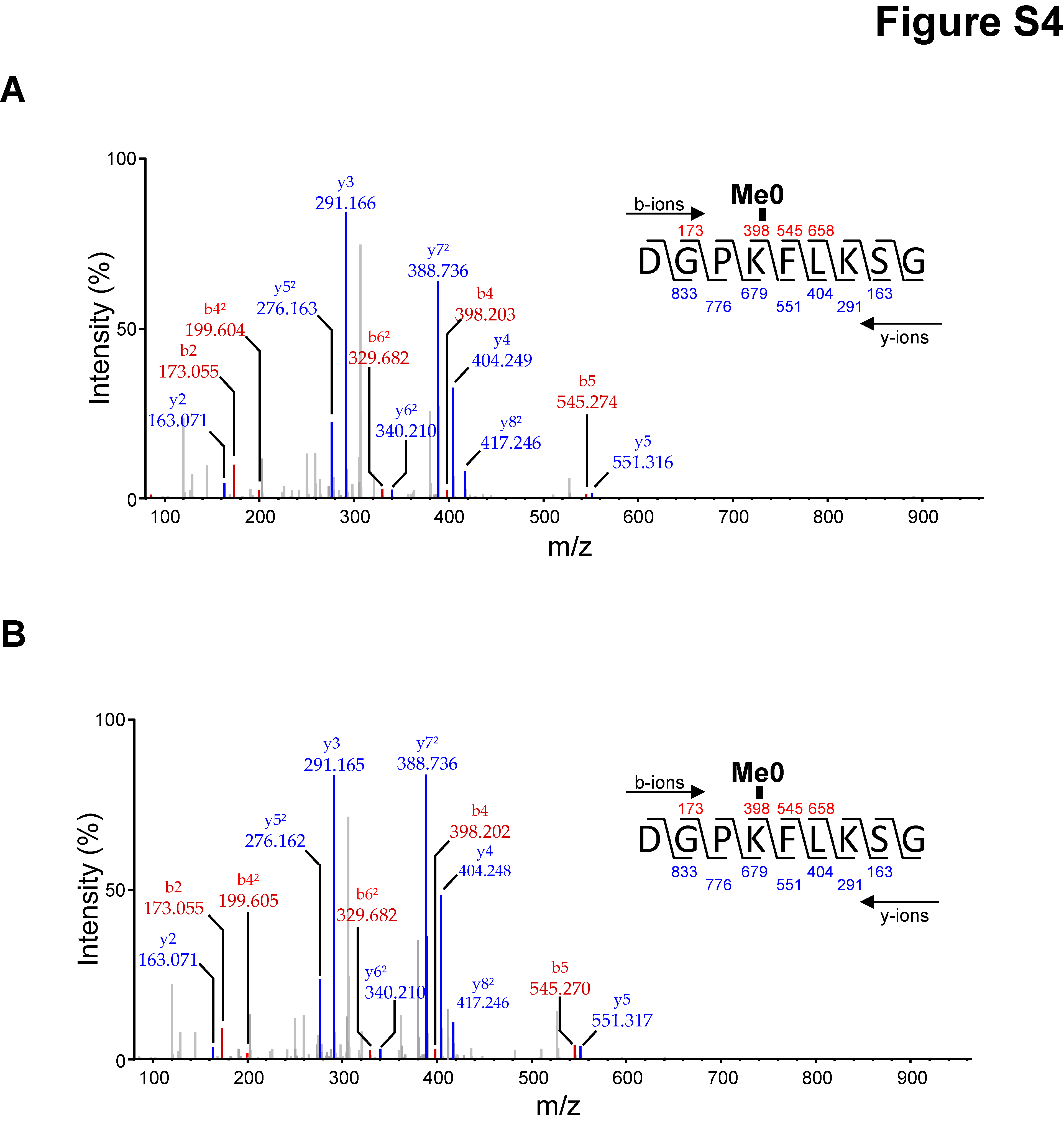

Supplement: S4 Fig — Annotated mass spectra from unmethylated peptide corresponding to aa 389–397 in human (A) and rabbit (B) eEF1A1. (TIF) [file pone.0131426.s004.tif]
