# Supplementary material for: Saccharomyces cerevisiae Eukaryotic Elongation Factor 1A (eEF1A) Is Methylated at Lys-390 by a METTL21-Like Methyltransferase
Source: PLoS One. 2015 Jun 26;10(6):e0131426. doi: 10.1371/journal.pone.0131426 (PMC4482628; doi:10.1371/journal.pone.0131426)
Supplement: S1 Table — (DOCX) [file pone.0131426.s005.docx]

**S1 Table. Plasmid constructs generated for and used in present study**

| Construct name | Source of ORF | Cloning method | Restriction sites used | Accession | SNP:s* | Reference |
| --- | --- | --- | --- | --- | --- | --- |
| pET28a-hsMETTL21A | HeLa cDNA | Restriction based | NdeI + BamH1 | AAL66295.1 | - | Jakobsson et al. 2013 |
| pGEX-6p-hsHSPA8 | IRATp970D1212D, 3920744 (IMAGE ID) | Restriction based | BamHI+XhoI | NP_006588.1 | - | Jakobsson et al. 2013 |
| pET28a-YNL024C | S. cerevisiae genomic DNA | Restriction based | NdeI+SacI | NM_001182863.1 | - | Kernstock et al. 2012 |
| pGEX-scSSA1 | S. cerevisiae genomic DNA | Restriction based | BamHI+XhoI | ref\|NP_009396.2\| | - | - |
| pGEX-scSSA3 | S. cerevisiae genomic DNA | Restriction based | EcoRI+XhoI | ref\|NP_009478.1\| | - | - |
| pGEX-scSSB2 | S. cerevisiae genomic DNA | Restriction based | BamHI+XhoI | gb\|EDN62613.1\| | - | - |
| pYES260-YNL024c | S. cerevisiae genomic DNA | Restriction based | NcoI + NotI | NM_001182863.1 | - | - |
| *Deviations in protein sequence between cloned constructs and best database hit. | | |  |  |  |  |
